# Bootstrap Confidence Intervals for Multiple Change Points Based on Two-Stage Procedures

**DOI:** 10.3390/e27050537

**Published:** 2025-05-17

**Authors:** Li Hou, Baisuo Jin, Yuehua Wu, Fangwei Wang

**Affiliations:** 1 Department of Statistics and Finance, University of Science and Technology of China, Hefei 230026, China; houli@ustc.edu.cn (L.H.); wfw0289@mail.ustc.edu.cn (F.W.); 2 Department of Mathematics and Statistics, York University, Toronto, ON M3J 1P3, Canada

**Keywords:** change point, bootstrap, confidence interval, OGA, sup-Wald-type test

## Abstract

This paper investigates the construction of confidence intervals for multiple change points in linear regression models. First, we detect multiple change points by performing variable selection on blocks of the input sequence; second, we re-estimate their exact locations in a refinement step. Specifically, we exploit an orthogonal greedy algorithm to recover the number of change points consistently in the cutting stage, and employ the sup-Wald-type test statistic to determine the locations of multiple change points in the refinement stage. Based on a two-stage procedure, we propose bootstrapping the estimated centered error sequence, which can accommodate unknown magnitudes of changes and ensure the asymptotic validity of the proposed bootstrapping method. This enables us to construct confidence intervals using the empirical distribution of the resampled data. The proposed method is illustrated with simulations and real data examples.

## 1. Introduction

Multiple change point detection is common in many applications, such as signal processing [1], medical diagnosis [2], industrial control [3], and oceanography [4]. For example, in the case of the detection of changes in one’s heart rates, the accurate identification of the change points can decompose the data into stationary segments, allowing clinicians to identify the behavior of the heart and diagnose diseases. The detection of multiple change points has important practical implications, which has prompted extensive research in the statistical community.

Implementing appropriate hypothesis testing is an important method for detecting multiple change points. Here are some examples. Ref. [5] proposed sup-Wald-type tests to test the null hypothesis of no change against alternative hypotheses containing an arbitrary number of changes to identify multiple structural changes in linear models. At the same time, the null hypothesis of *s* changes against s+1 changes is tested to determine the number of breakpoints. Later, ref. [6] developed dynamic programming principles to estimate multiple change points in linear regression. Ref. [7] proposed a genetic algorithm for detecting multiple breakpoints. However, these methods are very time-consuming. There is another line of research on multiple change point detection that transforms the problem into a variable selection problem. For example, ref. [8] used LASSO [9] to estimate the locations of multiple change points in a one-dimensional piecewise constant signal observed in white noise. Ref. [10] introduced a two-stage procedure based on adaptive LASSO [11], SCAD [12], and MCP [13] regularization methods that can simultaneously detect multiple change points in linear models. In addition, the two-stage procedure has been extended to accelerated failure time (AFT) models [14].

Despite the aforementioned developments, research on the inference for multiple change points remains limited. Under the null hypothesis, asymptotic [15] or approximate [16] distributions of test statistics have been derived, which enables quantifying uncertainty in the number of change points. For many testing procedures in change point analysis, the computation of critical values often relies on the asymptotic behavior of the test statistic under the null hypothesis. However, the convergence of the test statistic to its limiting distribution is often slow, and in some cases, the exact form of this distribution remains unknown. It has been noted that the bootstrap method is a computer-based statistical inference technique that can provide answers to a variety of statistical questions without relying on formulas. For example, ref. [17] proposed a bootstrap method in which the estimated error sequence is resampled with replacement to obtain confidence intervals for change points. Ref. [18] introduced an asymptotically valid confidence region for a single change point through the inversion of bootstrap tests. Ref. [19] studied the application of a circular overlapping block bootstrap method in the context of an at-most-one-change time series model with an abrupt change in the mean and dependent errors. In the field of a single change point estimation, the bootstrap technique has emerged as a valuable tool for approximating unknown probability distributions and the characteristics of change point estimators. These methods make it possible for us to provide confidence intervals for multiple change points. Ref. [20] addressed this problem in mean shift models by proposing the bootstrap construction of pointwise and uniform confidence intervals for multiple change points based on a moving sum procedure.

This paper aims to construct confidence intervals for multiple change points. By [10], the multiple change point detection problem can be formulated as a model selection problem. Before using bootstrapping, it is important to ensure that the estimates of the number of change points and the estimates of the within-segment parameters are consistent. However, regularization methods such as the LASSO and SCAD may suffer from the bias problem inherited from the penalty function [12,21]. Specifically, LASSO may select more irrelevant variables, leading to an overestimation of the number of change points. To alleviate this problem, we consider adopting the $L_{0}$-regularization method to achieve consistency between the estimates of the number of change points and their locations. Although the subset selection, while unbiased, is often computationally expensive, several optimization strategies and algorithms have been proposed to overcome the computational difficulties. For example, ref. [22] introduced an iterative algorithm called the orthogonal greedy algorithm (OGA) for high-dimensional regression models, which sequentially selects input variables to be included in the linear regression model and proposed a procedure named OGA + HDIC + Trim. OGA is a fast stepwise forward regression method that starts with a null model and adds predictors via component-wise linear least squares estimation. HDIC is a high-dimensional information criterion used to enter predictors into the model along the OGA path. The Trim method defines a subset to exclude additional irrelevant variables from OGA + HDIC. To construct confidence intervals for multiple change points, we proceed as follows. We first cut the data sequence into segments. Due to its model selection consistency and expected convergence properties, we apply OGA + HDIC + Trim to find data segments containing change points. We then utilize sup-Wald-type test statistics to locate them. Finally, we apply the bootstrap method to construct confidence intervals for multiple change points using the bootstrap estimated centered error sequence.

The main contributions of this paper are as follows. As described above, we first propose a two-stage procedure to detect multiple change points by using OGA + HDIC + Trim procedure and sup-Wald-type test statistics. In the first stage, we cut the data sequence into segments. In addition, we give the asymptotic distributions of the change point estimators under certain conditions. Based on this framework, we further explore the application of bootstrapping techniques in constructing confidence intervals for the change points and demonstrate the validity of the bootstrap method, i.e., the proposed bootstrap 100(1−α)%-confidence intervals asymptotically attain the coverage probability of 1−α for a given α∈(0,1) [20]. Last but not least, we illustrate the effectiveness and applicability of the proposed method through extensive simulation studies and a real data example, respectively.

The rest of this paper is organized as follows. Section 2 details the detection of multiple change points using the OGA + HDIC + Trim procedure and sup-Wald-type test statistics. Furthermore, Section 3 introduces the resampling bootstrap method for the change point estimators based on a two-stage procedure and Section 4 gives the theoretical properties of the proposed bootstrap method. Section 5 presents extensive simulation studies. Section 6 applies the proposed method to the seismograms of the 1982 Urakawa–Oki earthquake. Technical proofs of the main results are relegated to the Appendix A. In this paper, vectors and matrices are denoted in bold type.

## 2. Multiple Change Point Detection Based on Two-Stage Procedures

Assume that $(x_{i},y_{i}),i=1,\ldots ,n,$ satisfy the following linear regression model with *s* change points located at $a_{1}<\ldots <a_{s}$ as

(1) $$\begin{array}{cc} y_{i}\; & =x_{i}^{⊤}\left[\beta_{1}+\sum_{l=1}^{s}\delta_{l}I\left(a_{l}<i\leq n\right)\right]+\varepsilon_{i}\\ \; & =\left\{\begin{array}{cc} x_{i}^{⊤}\beta_{1}+\varepsilon_{i}, & \mathrm{if}\;1\leq i\leq a_{1},\\ x_{i}^{⊤}\left(\beta_{1}+\delta_{1}\right)+\varepsilon_{i}, & \mathrm{if}\;a_{1}<i\leq a_{2},\\ \cdots & \cdots \\ x_{i}^{⊤}\left(\beta_{1}+\sum_{l=1}^{s}\delta_{l}\right)+\varepsilon_{i}, & \mathrm{if}\;a_{s}<i\leq n, \end{array}\right. \end{array}$$

where *n* is the sample size, $x_{i}={\left(x_{i,1},\ldots ,x_{i,q}\right)}^{⊤}$ is a sequence of *q*-dimensional predictors; $\beta_{1}={\left(\beta_{1,1},\ldots ,\beta_{q,1}\right)}^{⊤}\neq 0$ is an unknown *q*-dimensional vector of regression coefficients; *s* is an unknown number of change points; $1<a_{1}<\ldots <a_{s}<n$ are unknown change points; $\delta_{l}={\left(\delta_{1,l},\ldots ,\delta_{q,l}\right)}^{⊤},l=1,\ldots ,s$, are unknown changes in regression coefficient vectors at change points; and $\varepsilon_{i}$’s are unobservable random errors.

The estimation of the regression coefficients $\beta_{1}$ and $(\beta_{1}+\sum_{l=1}^{j}\delta_{l}),j=1,\ldots s$ is hampered by the unknown parameters *s*, $a_{1},\ldots ,a_{s}$. In light of [23], we propose to replace *s* in (1) with a predetermined number of segments, denoted by $p_{n}$, to facilitate the estimation of the regression coefficients. This replacement allows us to identify the total number of change points. Specifically, we partition the data sequence into $p_{n}$ segments, where $p_{n}\to \infty$ as n→∞. This ensures that all segments excluding the first segment have length *m*, while the length of the first segment is $n-(p_{n}-1)m$. Let $Q_{1}=$$\left\{1,2,\ldots ,n-\left(p_{n}-1\right)m\right\}$ and $Q_{l}=\left\{n-\left(p_{n}-l+1\right)m+1,\ldots ,n-\left(p_{n}-l\right)m\right\},\;l=2,\ldots ,p_{n}$. See (3) for more details on segmentation. We assume that $m<\min(a_{j+1}-a_{j})/2$ for j=1,⋯,s, so that each segment $Q_{l}$, for $l=1,\cdots ,p_{n}$, contains at most one change point.

We denote the segment as $k_{j}$ if $a_{j}\in Q_{k_{j}}$. This partitioning results in the following model:

(2) $$\begin{array}{cc} y_{i} & =x_{i}^{⊤}\left[\beta_{1}+\sum_{l=2}^{p_{n}}d_{l}I\left(n-\left(p_{n}-l+1\right)m<i\leq n\right)-\omega_{i}\right]+\varepsilon_{i}, \end{array}$$

where

$$\left\{\begin{array}{cc} d_{k_{j}}=\delta_{j}\neq 0, & \;\mathrm{for}\;j=1,\ldots ,s,\\ d_{l}=0, & \;\mathrm{for}\;\mathrm{any}\;l\notin \left\{k_{1},\ldots ,k_{s}\right\}, \end{array}\right.$$

and $\omega_{i}=d_{k_{j}}I\left(i\in T_{j}\right)$ with $T_{j}=\left\{n-\left(p_{n}-k_{j}+1\right.\right.$) $\left.m+1,\ldots ,a_{j}\right\}$. $\omega_{i}=0$ for all $i\notin \cup_{j=1}^{s}Q_{k_{j}}$.

**Remark** **1.** *By the definition of m, any two segments also contain at most one change point. From* (2)*, it follows that*

$$\begin{array}{cc} y_{i} & =\left\{\begin{array}{cc} x_{i}^{⊤}\left[\beta_{1}+\sum_{l=1}^{j-1}\delta_{l}\right]+\varepsilon_{i}, & \mathrm{if}\;i\in Q_{k_{j}-1},\\ x_{i}^{⊤}\left[\beta_{1}+\sum_{l=1}^{j}\delta_{l}-\omega_{i}\right]+\varepsilon_{i}, & \mathrm{if}\;i\in Q_{k_{j}},\\ x_{i}^{⊤}\left[\beta_{1}+\sum_{l=1}^{j}\delta_{l}\right]+\varepsilon_{i}, & \mathrm{if}\;i\in Q_{k_{j}+1}. \end{array}\right. \end{array}$$

*Based on the partition, we can obtain least-squares estimates ${\hat{\gamma }}_{l}$ for $l=k_{j}-1,k_{j},k_{j}+1$ within each segment.*

*1.*  *When $a_{j}=n-\left(p_{n}-k_{j}\right)m$, the change point coincides with the pre-specified cut-point and $\omega_{i}=\delta_{j}$. The regression coefficients corresponding to the three segments are denoted by $\gamma_{l},l=k_{j}-1,k_{j},k_{j}+1$, and are equal to $\beta_{1}+\sum_{l=1}^{j-1}\delta_{l}$, $\beta_{1}+\sum_{l=1}^{j-1}\delta_{l}$, and $\beta_{1}+\sum_{l=1}^{j}\delta_{l}$, respectively. The segment $Q_{k_{j}}$ that contains the change point $a_{j}$ can be identified by ${\hat{\gamma }}_{k_{j}+1}-{\hat{\gamma }}_{k_{j}}\neq 0$.*
*1.*  *When $a_{j}<n-\left(p_{n}-k_{j}\right)m$, due to the existence of $\omega_{i}$, the linear regression model is misspecified on $Q_{k_{j}}$, causing ${\hat{\gamma }}_{k_{j}}$ to converge to the pseudo-true value $\gamma_{k_{j}}$ under model misspecification [24,25]. Since $\gamma_{k_{j}}$ is different from both $\beta_{1}+\sum_{l=1}^{j-1}\delta_{l}$ and $\beta_{1}+\sum_{l=1}^{j}\delta_{l}$, $Q_{k_{j}}$ can be determined by the following: ${\hat{\gamma }}_{k_{j}}-{\hat{\gamma }}_{k_{j}-1}\neq 0$ and ${\hat{\gamma }}_{k_{j}+1}-{\hat{\gamma }}_{k_{j}}\neq 0$.*

According to Remark 1, we reformulate the change-point detection problem as a high-dimensional variable selection task by constructing differences between coefficients. Therefore, (2) can be rewritten in matrix form as follows:

(3) $$y_{n}=X_{n}\theta +e_{n}+\varepsilon_{n},$$

where $y_{n}={\left(y_{1},y_{2},\ldots ,y_{n}\right)}^{⊤}$,

$$X_{n}=\left(X^{\left(1\right)},\ldots ,X^{\left(p_{n}\right)}\right)=\left(\begin{array}{cccc} X_{\left(1\right)} & 0 & \cdots & 0\\ X_{\left(2\right)} & X_{\left(2\right)} & \cdots & 0\\ \vdots & \vdots & \ddots & \vdots \\ X_{\left(p_{n}\right)} & X_{\left(p_{n}\right)} & \cdots & X_{\left(p_{n}\right)} \end{array}\right),$$

$X_{\left(1\right)}={\left(x_{1},\cdots ,x_{n-(p_{n}-1)m}\right)}^{⊤}$ and $X_{\left(j\right)}={\left(x_{n-(p_{n}-j+1)m+1},\ldots ,x_{n-(p_{n}-j)m}\right)}^{⊤}$. In addition, the definitions of $\theta ={\left(\theta_{1}^{⊤},\ldots ,\theta_{p_{n}}^{⊤}\right)}^{⊤}={\left(\beta_{1}^{⊤},{\left(\gamma_{2}-\beta_{1}\right)}^{⊤},\ldots ,{\left(\gamma_{p_{n}}-\gamma_{p_{n}-1}\right)}^{⊤}\right)}^{⊤}$, $\varepsilon_{n}={\left(\varepsilon_{1},\ldots ,\varepsilon_{n}\right)}^{⊤}$, and $e_{n}$ represents the artificial *n*-dimensional error due to model misspecification, whose elements are equal to zero for all $i\notin \cup_{j=1}^{s}Q_{k_{j}}$.

It can be seen that $\theta_{l}=0$ if $l\neq 1,k_{j},$ or $k_{j}+1$ for j=1,…,s. Let $A_{n}=\left\{1,k_{1},k_{1}+1,k_{2},k_{2}+1,\ldots ,k_{s},k_{s}+1\right\}$. It is important to note that $\theta_{l}=0_{q\times 1}$ is evident for all $l\notin A_{n}$. Therefore, the estimation of $A_{n}$ comes down to identifying the non-zero elements from θ, which is the focus of the subsequent subsections.

### 2.1. Segment Selection

By the definition of *m*, each segment $Q_{k_{j}}$ contains a change point. In this subsection, our goal is to select segments $Q_{k_{j}}$ for j=1,⋯,s. From the above statement, we can see that selecting all non-zero elements of θ can yield the estimation of $A_{n}$ effectively. Therefore, after the cutting stage, the problem of detecting multiple change points is reduced to a variable selection problem in high-dimensional scenarios. We use the OGA + HDIC + Trim method for the segment selection.

We rewrite the model (3) as follows:

(4) $$y_{n}=\sum_{j=1}^{r_{n}}z_{j}\theta_{j}+{\tilde{\varepsilon }}_{n},$$

where $r_{n}=p_{n}\cdot q$, $\left\{z_{1},\ldots ,z_{r_{n}}\right\}$ are all the column vectors of $x_{n}$, ${\tilde{\varepsilon }}_{n}=e_{n}+\varepsilon_{n}$ and $\theta ={\left(\theta_{1},\cdots ,\theta_{r_{n}}\right)}^{⊤}$. Without loss of generality, replace $y_{n}$ by $y_{n}-y_{n}^{⊤}1_{n}$ and $z_{j}$ by $z_{j}-z_{j}^{⊤}1_{n}$. Define ${\hat{\sigma }}_{J_{d}}^{2}=n^{-1}y_{n}^{⊤}\left(I_{n}-H_{J_{d}}\right)y_{n}$, where $H_{J_{d}}$ is the orthogonal projection matrix onto the linear span of $\left\{z_{j},j\in J_{d}\right\}$. For convenience, denote $H_{\emptyset }=0$. The proposed OGA+HDIC+Trim algorithm is outlined in Algorithm 1.
**Algorithm 1** OGA + HDIC + Trim**Require:** response vector $y_{n}\in R^{n}$, regressor matrix $X_{n}\in R^{n\times r_{n}}$.
 Initialzation: set d=0, $r^{\left(0\right)}=y_{n}$ and ${\hat{J}}_{0}=\emptyset$.
 **While** $d=0,\ldots ,D_{n}$ **do**
    Compute ${\hat{j}}_{d+1}=\arg\max_{1\leq j\leq r_{n},j\notin {\hat{J}}_{d-1}}|z_{j}^{⊤}r^{\left(d\right)}|/(n^{1/2}∥z_{j}∥)$ and update ${\hat{J}}_{d+1}={\hat{J}}_{d}⋃{\hat{j}}_{d+1}$;
    Compute $z_{{\hat{j}}_{d+1}}^{⊥}$ via $z_{{\hat{j}}_{d+1}}^{⊥}=\left(I_{n}-\sum_{\ell =1}^{d}H_{{\hat{j}}_{\ell }}^{⊥}\right)z_{{\hat{j}}_{d+1}}$, where $H_{{\hat{j}}_{\ell }}^{⊥}=z_{{\hat{j}}_{\ell }}z_{{\hat{j}}_{\ell }}^{⊤}/{∥z_{{\hat{j}}_{\ell }}∥}^{2}$;
    Compute $r^{\left(d+1\right)}$ via $r^{\left(d+1\right)}=\left(I_{n}-H_{{\hat{j}}_{d+1}}^{⊥}\right)r^{\left(d\right)}$.
 **end**
    Compute the minimum of HDIC via(5)$$\begin{array}{cc} \mathrm{HDIC}({\hat{J}}_{d}) & =\log{\hat{\sigma }}_{{\hat{J}}_{d}}^{2}+♯\left({\hat{J}}_{d}\right)c_{n}\log\left(r_{n}\right)/n,\\ {\hat{d}}_{n} & =\arg\min_{1\leq d\leq D_{n}}\mathrm{HDIC}\left({\hat{J}}_{d}\right). \end{array}$$
 **return** ${\hat{J}}_{n}$ via$${\hat{J}}_{n}=\left\{\begin{array}{cc} \{{\hat{j}}_{\ell }:\mathrm{HDIC}\left({\hat{J}}_{d}-\left\{{\hat{j}}_{\ell }\right\}\right)>\mathrm{HDIC}\left({\hat{J}}_{d}\right),\;1\leq \ell \leq {\hat{d}}_{n}\}, & \;\mathrm{if}\;{\hat{d}}_{n}>1;\\ \{{\hat{j}}_{1}\}, & \;\mathrm{if}\;{\hat{d}}_{n}=1. \end{array}\right.$$


In this context, the value of $D_{n}$ denotes the maximum number of iterations, and the convergence rate theory of OGA in [22] shows that $D_{n}=O\left(n^{1/2}{\left(\log r_{n}\right)}^{-1/2}\right)$. The parameter $c_{n}$ satisfies the conditions $c_{n}\to \infty$ and $c_{n}\log r_{n}=o\left(n^{1-2\gamma }\right)$, where γ∈[0,1). Let $\hat{\theta }={\left({\hat{\theta }}_{1}^{⊤},\ldots ,{\hat{\theta }}_{p_{n}}^{⊤}\right)}^{⊤}$ be the estimate obtained by applying the OGA+HDIC+Trim procedure. Therefore, we derive an estimate of $A_{n}$ from ${\hat{J}}_{n}$ as ${\hat{A}}_{n}=\left\{l:{\hat{\theta }}_{l}\neq 0,l=1,\ldots ,p_{n}\right\}.$ Denote

(6) $${\hat{C}}_{n}=\left\{l:l\in {\hat{A}}_{n},l+1\notin {\hat{A}}_{n},l=2,\ldots ,p_{n}+1\right\}=\left\{{\hat{k}}_{1},\ldots ,{\hat{k}}_{\hat{s}}\right\},$$

where ${\hat{k}}_{1}<\cdots <{\hat{k}}_{\hat{s}}$. It is clear that if $l+1\notin {\hat{A}}_{n},l\in {\hat{A}}_{n}$ and $l-1\in {\hat{A}}_{n}$, then $l\in {\hat{C}}_{n}$ and $l-1\notin {\hat{C}}_{n}$. Let $Q_{\left(j\right)}=Q_{k_{j}}\cup Q_{k_{j}+1}$. It only includes the change point $a_{j}$ since $m<\min_{j}\left(a_{j+1}-a_{j}\right)/2$, and it ensures that no change point overlaps with the cut-point of $Q_{\left(j\right)}$. By following the steps outlined above and considering that the Wald test cannot detect the location of the change point at the partition boundary, we derive the following selected segments:

(7) $${\hat{Q}}_{\left(j\right)}={\hat{Q}}_{{\hat{k}}_{j}}⋃{\hat{Q}}_{{\hat{k}}_{j}+1}=\left\{n-\left(p_{n}-{\hat{k}}_{j}+1\right)m+1,n-\left(p_{n}-{\hat{k}}_{j}-1\right)m\right\},$$

where ${\hat{Q}}_{{\hat{k}}_{j}}=\left\{n-\left(p_{n}-{\hat{k}}_{j}+1\right)m+1,\cdots ,n-\left(p_{n}-{\hat{k}}_{j}\right)m\right\}$.

### 2.2. Refining

By Theorem 1 in Section 4, $\hat{s}$ converges to *s* as n→∞. Hence, we assume that for a large *n*, there exists ${\hat{Q}}_{\left(j\right)}$ such that $a_{j}\in {\hat{Q}}_{\left(j\right)}$. We now show how to estimate this change point. Note that for $i\in {\hat{Q}}_{\left(j\right)}=\left\{n-\left(p_{n}-{\hat{k}}_{j}+1\right)m+1,n-\left(p_{n}-{\hat{k}}_{j}-1\right)m\right\}$, we have

(8) $$y_{i}=x_{i}^{⊤}\left[\beta_{j}+\delta_{j}I\left(a_{j}<i\leq n_{j}^{\left(r\right)}\right)\right]+\varepsilon_{i},\;n_{j}^{\left(l\right)}\leq i\leq n_{j}^{\left(r\right)},$$

where $n_{j}^{\left(l\right)}=n-\left(p_{n}-{\hat{k}}_{j}+1\right)m+1$ and $n_{j}^{\left(r\right)}=n-\left(p_{n}-{\hat{k}}_{j}-1\right)m$. Here, $\delta_{j}=\beta_{j+1}-\beta_{j}$ and $\beta_{j}$ and $\beta_{j+1}$ are unknown *q*-dimensional vectors of the regression coefficients on the line segment $\left\{n_{j}^{\left(l\right)},a_{j}\right\}$ and $\left\{a_{j},n_{j}^{\left(r\right)},\right\}$, respectively. $n_{j}^{\left(r\right)}-n_{j}^{\left(l\right)}+1=2m$.

We compute the sup-Wald test statistics [26] and estimate $a_{j}$ by

(9) $${\hat{a}}_{j,h}=\arg\max_{h}{\hat{\delta }}_{j;h}^{⊤}\left(Z_{j,h}^{⊤}M_{j}Z_{j,h}\right){\hat{\delta }}_{j;h},\;n_{j}^{\left(l\right)}+q<h<n_{j}^{\left(r\right)}-q,$$

where $Z_{j,h}={\left(0,\cdots ,0,x_{h+1},x_{h+2},\cdots ,x_{n_{j}^{\left(r\right)}}\right)}^{⊤}$, $X_{j}={\left(x_{n_{j}^{\left(l\right)}},x_{n_{j}^{\left(l\right)}+1}.\cdots ,x_{n_{j}^{\left(r\right)}}\right)}^{⊤}$, and $M_{j}=I_{n}-X_{j}{\left(X_{j}^{⊤}X_{j}\right)}^{-1}x_{j}^{⊤}$. We also obtain the estimates $\left({\hat{\beta }}_{j;h},{\hat{\delta }}_{j;h}\right)$ of $\left(\beta_{j},\delta_{j}\right)$ by regressing $y_{\left(j\right)}={\left(y_{n_{j}^{\left(l\right)}},\cdots ,y_{n_{j}^{\left(r\right)}}\right)}^{⊤}$ on $X_{j}$ and $Z_{j,h}$, respectively. The limiting behavior of ${\hat{a}}_{j,h}$ is given in Section 4.

Since the multiple change points are not dense in the data sequence, without loss of generality, we assume that $m<\min(a_{j+1}-a_{j})/2,$j=1,…,s−1, so that each segment $Q_{\left(j\right)}$ contains at most one change point. Note that if *m* is too small, it leads to inconsistency in estimating the regression parameters and increases the computational time. Therefore, we need to avoid choosing too small a value for *m*. To address this issue, we define $m=\lceil c_{0}\sqrt{n}\rceil$ according to Theorem 2 in Section 3, where $c_{0}$ serves as a tuning parameter, and ⌈·⌉ is the ceiling function. We study the range of values of $c_{0}$ on the interval [0.1,1.5]. The final value of *m* is determined using the Bayesian Information Criterion (BIC) as follows:

$$\hat{m}=\arg\min_{m}\log\left\{\sum_{i=1}^{n}{\left(y_{i}-x_{i}^{⊤}\left[{\hat{\beta }}_{1,h}+\sum_{j=1}^{\hat{s}}{\hat{\delta }}_{j,h}I\left({\hat{a}}_{j,h}<i\leq n\right)\right]\right)}^{2}\right\}+\hat{s}\cdot q\log n.$$

## 3. Bootstrap Confidence Intervals for Multiple Change Points

In this section, we construct bootstrap confidence intervals for multiple change points. It helps us to study the behavior of multiple change points in a linear regression model. Obviously, the two-stage procedure inherently involves the quantification of uncertainty in the number of change points and their respective locations.

We define the estimated residuals ${\hat{\varepsilon }}_{i}$ and the centered residuals ${\tilde{\varepsilon }}_{i}$ as follows:

(10) $$\begin{array}{cc} {\hat{\varepsilon }}_{i} & =y_{i}-x_{i}^{⊤}\left[{\hat{\beta }}_{1,h}+\sum_{l=1}^{\hat{s}}{\hat{\delta }}_{l,h}I\left({\hat{a}}_{l,h}<i\leq n\right)\right],\\ {\tilde{\varepsilon }}_{i} & ={\hat{\varepsilon }}_{i}-\frac{1}{{\hat{a}}_{j,h}-{\hat{a}}_{j-1,h}}\sum_{l={\hat{a}}_{j-1,h}+1}^{{\hat{a}}_{j,h}}{\hat{\varepsilon }}_{l},\;{\hat{a}}_{j-1,h}<i\leq {\hat{a}}_{j,h}, \end{array}$$

where ${\hat{a}}_{0,h}=1$ and ${\hat{a}}_{\hat{s}+1,h}=n$. Let $\varepsilon_{{\hat{a}}_{j-1,h}+1}^{*},\ldots ,\varepsilon_{{\hat{a}}_{j,h}}^{*}$ be independently and identically distributed (i.i.d.) random variables sampled from the empirical distribution function of $\left\{{\tilde{\varepsilon }}_{{\hat{a}}_{j-1,h}+1},\ldots ,{\tilde{\varepsilon }}_{{\hat{a}}_{j,h}}\right\}$. We then consider the bootstrap observations defined as follows:

(11) $$\begin{array}{cc} y_{i}^{*}\; & =x_{i}^{⊤}\left[{\hat{\beta }}_{1,h}+\sum_{l=1}^{\hat{s}}{\hat{\delta }}_{l,h}I\left({\hat{a}}_{l,h}<i\leq n\right)\right]+\varepsilon_{i}^{*},\;i=1,\ldots ,n, \end{array}$$

where $\varepsilon_{i}^{*}$ represents the bootstrapped version of the residuals. To obtain an approximation to the distribution of ${\hat{a}}_{j;h}$ in (9), a bootstrap statistic of the estimate is defined as

(12) $${\hat{a}}_{j^{*};h_{b}}^{*}=\arg\max_{h_{b}}{\hat{\delta }}_{j^{*};h_{b}}^{*⊤}\left(z_{j^{*},h_{b}}^{⊤}M_{j}Z_{j^{*},h_{b}}\right){\hat{\delta }}_{j^{*};h_{b}}^{*},\;j^{*}=1,\ldots ,{\hat{s}}^{*},$$

where the number of change points ${\hat{s}}^{*}$ and the ${\hat{C}}_{n}^{*}$ are obtained by performing OGA on $y_{n}^{*}={\left(y_{1}^{*},\ldots ,y_{n}^{*}\right)}^{⊤}$ as in (6). The data sequence segmentation remains unchanged. In this case, $Z_{j^{*},h_{b}}={\left(0,\ldots ,0,x_{h_{b}+1},\ldots ,x_{n_{j^{*}}^{\left(r\right)}}\right)}^{⊤}$, and $\left({\hat{\beta }}_{j^{*};h_{b}}^{*},{\hat{\delta }}_{j^{*};h_{b}}^{*}\right)$ represents the least-squares estimates obtained by regressing $y_{\left(j^{*}\right)}^{*}={\left(y_{n_{j^{*}}^{\left(l\right)}}^{*},\ldots ,y_{n_{j^{*}}^{\left(r\right)}}^{*}\right)}^{⊤}$ on $X_{j^{*}}$ and $Z_{j^{*},h_{b}}$. Next, we describe the construction of bootstrap CIs for multiple change points.

1. We generate a bootstrap sample ${\left(y_{1}^{*},\ldots ,y_{n}^{*}\right)}^{⊤}$ by randomly sampling residuals ${\left(\varepsilon_{1}^{*},\ldots ,\varepsilon_{n}^{*}\right)}^{⊤}$ from the set $\left\{{\tilde{\varepsilon }}_{1},\ldots ,{\tilde{\varepsilon }}_{n}\right\}$ as in (11);
2. We apply the two-stage procedure and compute the local maximizer obtained as in (12) for each estimated segment;
3. For a given bootstrap sample size *B*, we repeat Steps 1-2 *B* times and record ${\hat{a}}_{j^{*};h_{b}}^{*(b)}$, $j^{*}=1,\cdots ,{\hat{s}}^{*}$, where b=1,…,B.

Therefore, the bootstrap-based approximation for the change point $a_{j}$ can be constructed. Generally, for any α∈(0,1), the bootstrap 100(1−α)% confidence interval for the change point $a_{j}$ is given by the following:

(13) $${\mathrm{CIs}}_{j}\left(\alpha \right)=\left[{\hat{a}}_{j,h}+q_{U}\left(\alpha /2\right),{\hat{a}}_{j,h}+q_{L}\left(\alpha /2\right)\right],$$

where $q_{U}\left(\alpha /2\right)=\sup\left\{x;\frac{1}{B}\sum_{b=1}^{B}I\left({\hat{a}}_{j^{*},h_{b}}^{*(b)}-{\hat{a}}_{j;h}\leq x\right)\leq \alpha /2\right\}$, and $q_{L}\left(\alpha /2\right)=\inf\left\{x;\frac{1}{B}\sum_{b=1}^{B}I\left({\hat{a}}_{j^{*},h_{b}}^{*(b)}-{\hat{a}}_{j;h}\geq x\right)\geq 1-\alpha /2\right\}.$

If ${\hat{C}}_{n}\subseteq {\hat{C}}_{n}^{*}$, then ${\hat{a}}_{j;h_{b}}^{*}$ is an estimate of ${\hat{a}}_{j;h}$ for $j=1,\ldots ,\hat{s}$. If some elements of ${\hat{C}}_{n}$ are not in the set ${\hat{C}}_{n}^{*}$, then ${\hat{a}}_{j;h}$ has no corresponding bootstrap estimate for some *j*. Hence, the bootstrap CI of ${\hat{a}}_{j;h}$ is constructed using $\left\{{\hat{a}}_{j;h_{b}}^{*(b)}:{\hat{a}}_{j;h_{b}}^{*(b)}\notin \emptyset ,b=1,\ldots ,B^{*}\right\}$ instead of $\left\{{\hat{a}}_{j^{*};h_{b}}^{*(1)},\ldots ,{\hat{a}}_{j^{*};h_{b}}^{*(B)}\right\}$ in (13), which yields

(14) $${\mathrm{CIs}}_{j}^{*}\left(\alpha \right)=\left[{\hat{a}}_{j,h}+q_{U}^{*}\left(\alpha /2\right),{\hat{a}}_{j,h}+q_{L}^{*}\left(\alpha /2\right)\right],$$

where

$$q_{U}^{*}\left(\alpha /2\right)=\sup\left\{x:\frac{1}{B^{*}}\sum_{b=1}^{B^{*}}I\left({\hat{a}}_{j,h_{b}}^{*(b)}-{\hat{a}}_{j;h}\leq x\right)\leq \alpha /2\right\},$$

and

$$q_{L}^{*}\left(\alpha /2\right)=\inf\left\{x:\frac{1}{B^{*}}\sum_{b=1}^{B^{*}}I\left({\hat{a}}_{j,h_{b}}^{*(b)}-{\hat{a}}_{j;h}\geq x\right)\geq 1-\alpha /2\right\}.$$

## 4. Theoretical Validity of the Bootstrap Confidence Intervals

To investigate the performance of the bootstrap CIs for multiple change points, we make the following assumptions.

**Assumption** **1.** *If s⩾1, then $a_{j}/n\to \tau_{j}>0$ for 1≤j≤s. Furthermore, if s⩾2, then $\min_{1\leq j\leq s-1}\left(\tau_{j+1}-\tau_{j}\right)>0$.*

**Assumption** **2.** *$\left\{\varepsilon_{i},\;i=1,2,\ldots ,n\right\}$ is a sequence of independently and identically distributed random variables with $E(\varepsilon_{i})=0$ and $E\left(\varepsilon_{i}^{2}\right)=\sigma^{2}$.*

**Assumption** **3.** *$a_{j}-n_{j}^{\left(l\right)}=\left\lceil 2\tau_{j}m\right\rceil$, where $\tau_{j}\in \left(0,1\right)$ and ⌈·⌉ is the ceilling function.*

**Assumption** **4.** *$\sup_{(t_{2}-t_{1})\geq 1}∥\sum_{i=t_{1}}^{t_{2}}x_{i}x_{i}^{⊤}/\left(t_{2}-t_{1}\right)∥$ is stochastically bounded. $\varepsilon_{i}$ is independent of the regressor $x_{j}$ for all i and j.*

**Assumption** **5.** *$\delta_{j}\to 0$, and $\delta_{j}^{-1}{\left(2m\right)}^{-1/2+\alpha }=o\left(1\right)$ for some α∈(0,1/2) as n→∞.*

By Assumption 1, it follows that $m/(a_{j+1}-a_{j})\to 0$, i.e., there is at most one change point in each segment for a large *n*. Assumption 2 follows that the residuals are independent and identically distributed and justifies the use of bootstrapping to estimate the central error, helping generate the sample distribution of the change points. Assumption 3 assumes that the shift point is bounded from the endpoints for asymptotic purposes. Assumption 4 requires that there is enough data around the change point and at the beginning and end of the sample so that the change point can be identified. The asymptotic distribution of ${\hat{a}}_{j,h}$ depends on various unknown quantities, with the magnitude of the change $\delta_{j}$ being the most significant. Assumption 5 is the minimum signal amplitude of the regression coefficient in the high-dimensional setting. The shift amplitude cannot be too small; otherwise, the change point will not be identified. Based on this, we give the necessary Assumption 5. Next, we establish the consistency of the number of change points $\hat{s}$ and the change point estimator ${\hat{a}}_{j,h}$. The following theorem provides the consistency of the estimated number of change points.

**Theorem** **1.** *Suppose that m→∞, $p_{n}\to \infty$, $D_{n}=O\left({\left(n/\log\left(r_{n}\right)\right)}^{1/2}\right)$, and $\log(r_{n})/n\to 0$ as n→∞. Under Assumptions 1–5, we have*

(15) $$\begin{array}{cc} \; & \lim_{n\to \infty }P\left(\hat{s}=s\right)=1;\\ \; & \lim_{n\to \infty }P\left(a_{j}\in {\hat{Q}}_{\left(j\right)},j=1,\ldots ,s|\hat{s}=s\right)=1, \end{array}$$

*where $\hat{s}$ and ${\hat{k}}_{j},j=1,\ldots ,\hat{s}$ are given in* (6)*.*

Theorem 1 extends Theorem 4 in [22] to the multiple change points detection case. The consistency and asymptotic distribution of change points estimators are given below.

**Theorem** **2.** *If $\sum_{i=t_{1}}^{t_{2}}x_{i}x_{i}^{⊤}/\left(t_{1}-t_{2}\right)\to_{p}V$ as $t_{2}-t_{1}\to \infty$, under Assumptions 1–4, we have that when n→∞, ${\hat{a}}_{j,h}-a_{j}=O_{p}(∥\delta_{j}{∥}^{-2})$ and*

$$\frac{\delta_{j}^{⊤}V\delta_{j}}{\sigma^{2}}\left({\hat{a}}_{j,h}-a_{j}\right)\to_{d}\arg\max\{W\left(c\right)-|c|/2:c\in R\},$$

*where V is a strictly positive definite matrix, {W(c):c∈R} is a two-sided Wiener process, and $\delta_{j}$ is a fixed value or satisfies $\delta_{j}\to 0$, as specified in Assumption 5.*

Subsequently, we establish the validity of the bootstrap CIs in (14). For future reference, we define some notations here. Any symbol with a superscript ∗ denotes an object under the bootstrap probability measure, rather than the original measure used in some of the other sections. For example, $E^{*}\left(\cdot \right)$ denotes the conditional expectation with respect to the bootstrap probability measure conditional on the original data. Similarly, $P^{*}\left(\cdot \right)$ denotes the conditional probability under the bootstrap measure.

**Theorem** **3.** *Under the assumptions of Theorem 2, we have*

$$\underset{x\in R}{\sup}\left|P^{*}\left({\hat{a}}_{j,h_{b}}^{*}-{\hat{a}}_{j,h}\leq x\right)-P\left({\hat{a}}_{j,h}-a_{j}\leq x\right)\right|\to_{p}0.$$

The proofs of Theorems 2 and 3 are given in Appendix A.

Combining Theorem 3 with Theorems 1 and 2 establishes the validity of the bootstrap method for multiple change points.

**Corollary** **1.** *Under Assumptions 1–5, we have that, as n→∞,*

$$\underset{x\in R}{\sup}\left|P^{*}\left(\cap_{j=1}^{s}\left\{{\hat{a}}_{j,h_{b}}^{*}-{\hat{a}}_{j,h}\leq x\right\}\right)-P\left(\cap_{j=1}^{s}\left\{{\hat{a}}_{j,h}-a_{j}\leq x\right\}\right)\right|\to_{p}0.$$

Since $P\left(a_{j}\in {\mathrm{CIs}}_{j}^{*}\left(\alpha \right)\right)\to 1-\alpha$ for each j=1,…,s, by the Bonferroni correction, we have $P\left(\cap_{j=1}^{s}\left\{a_{j}\in {\mathrm{CIs}}_{j}^{*}\left(\alpha /s\right)\right\}\right)\to 1-\alpha$. The asymptotic validity of the proposed bootstrap CIs in (14) follows.

## 5. Simulation

In this section, we first present the simulation results for change point detection given in Section 2 and compare them with the two-stage multiple change point detection procedure involving LASSO (TSMCD_lasso_) by [10]. We also construct confidence intervals using the bootstrap method proposed in Section 3. We denote the CIs in (14) by bootstrap_oga,wald_.

### 5.1. Detection of Multiple Change Points

**Figure 1:**
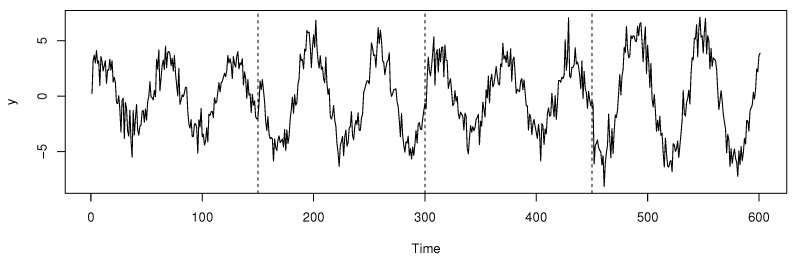
Simulated regression data with change points indicated by dotted lines.

We consider the simulation setting where the change points $a_{j}$, j=1,2,3 are, respectively, 150, 300, and 450, respectively, and generate data from the model

(16) $$\begin{array}{cc} y_{t}= & 2\cos\left(t\pi /30\right)+2\sin\left(t\pi /30\right)+0.1y_{t-1}\\ \; & +\left(-3\cos\left(t\pi /30\right)+\sin\left(t\pi /30\right)+0.2y_{t-1}\right)I_{\left(150,600\right]}\left(t\right)\\ \; & +\left(2\cos\left(t\pi /30\right)-0.3y_{t-1}\right)I_{\left(300,600\right]}\left(t\right)\\ \; & +\left(2\cos\left(t\pi /30\right)+2\sin\left(t\pi /30\right)\right)I_{\left(450,600\right]}\left(t\right)+\varepsilon_{t}, \end{array}$$

where $\varepsilon_{1},\ldots ,\varepsilon_{n}$ are independent and follow the standard normal distribution. The simulated data are shown in Figure 1. In this model, $\left\{y_{t},\;t=1,\cdots ,600\right\}$ represents a periodic autocorrelation sequence with a period of 30 and an autocorrelation order of 1.

We perform 1000 Monte Carlo simulations for multiple change point estimation in Table 1. According to [22], we take $c_{n}=2$ in (5), similar to AIC. We focus on counting the number of events for which $\left\{|{\hat{a}}_{j}-a_{j}|\leq 5\right\}$. The percentage of the correct identifications of all change points, denoted as $c_{\mathrm{all}}\left(\%\right)$, reflects the proportion of replicates for which $|{\hat{a}}_{j}-a_{j}|\leq 5$ for all *j*. In addition, $c_{j}\left(\%\right)$ represents the proportion of replicates for which $|{\hat{a}}_{j}-a_{j}|\leq 5$. The mean and standard error of the estimated change points are calculated for the replicates for which the difference between the estimated change points and the true value is less than or equal to 50 (i.e., $|{\hat{a}}_{j}-a_{j}|\leq 50$).

From Table 1, we can see that TSP_oga,wald_ generally outperforms TSMCD_lasso_ in terms of yielding a correct identification rate. It is noteworthy that TSMCD_lasso_ performs significantly worse than TSP_oga,wald_ in identifying all change points, especially at $a_{1}$, as evidenced by the lower $c_{1}$ value. TSP_oga,wald_ and TSMCD_lasso_ show comparable estimation accuracy in terms of the mean and standard error (SE) of the estimated change points.

### 5.2. Bootstrap CIs

All results are based on 500 realizations in the simulation setting, and we use B=500 to give the corresponding bootstrap CIs. We assume the confidence level is (1−α)∈{0.9,0.95}. For each *j*, the coverage of the bootstrap CI is calculated as the proportion of simulated realizations where ${\mathrm{CIs}}_{j}^{*}\left(\alpha \right)$ contains $a_{j}$. For all *j*, the coverage of the bootstrap CIs is calculated as the proportion of simulated realizations, where $\cup_{j=1}^{s}{\mathrm{CIs}}_{j}^{*}\left(\alpha /s\right)$ contains all $a_{j}$.

Table 2 confirms the effectiveness of our bootstrap method, as the empirical coverage probability for each $a_{j}$ is close to the nominal level. The overall coverage of the bootstrap confidence intervals for all *j* is slightly higher than the nominal level, which may be due to the complexity of multiple change point detection. The average computational time for the bootstrap_oga,wald_ procedure is 1.44 min per Monte Carlo replication, as measured on an Intel(R) Core(TM) i9-14900K processor (3.20 GHz) with 64 GB of RAM.

## 6. Empirical Application

**Figure 2:**
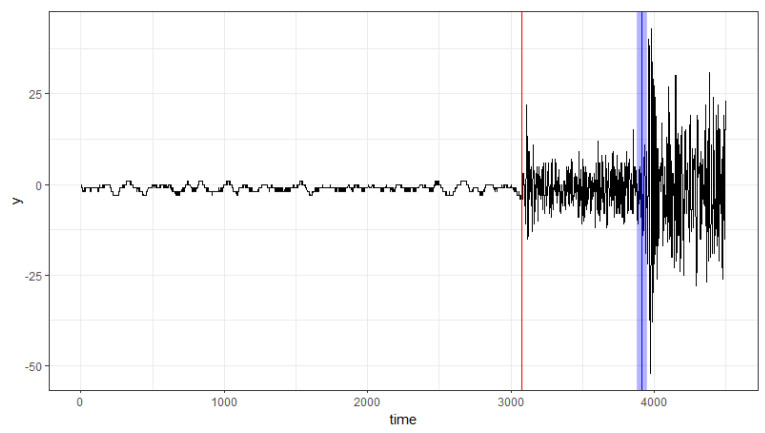
Change points estimated by our method (vertical lines), with shaded areas representing the 95% confidence intervals around the change points. The results for the two change points are shown in red and blue, respectively.

In this section, we illustrate the proposed method by an application to the east–west component of seismograms, recorded at Iwanai station during the first foreshock of the Urakawa–Oki earthquake in 1982. This dataset has been previously studied by [27]. The time series data are analyzed using autoregressive models (AR) of an order of 5. The estimates and visualization of the 95% confidence level bootstrap CIs are presented in Table 3 and Figure 2, respectively.

It can be seen from Table 3 that change points are detected at 3074 and 3914. In [27], they found the estimated change points to be 3079 and 3929. It is noteworthy that [27]s’ results and ours are close. In geology, these two change points represent the arrival times of P-waves and S-waves, which are two types of seismic waves. From Figure 2, it is clear that the confidence interval (CI) of the first change point is narrower than that of the second change point.

This example demonstrates the applicability and effectiveness of our method in detecting change points in seismic data. By accurately identifying the locations of structural faults, we can gain insights into the underlying geological processes and improve our understanding of seismic events.

## 7. Conclusions

This paper effectively addresses the bias issues often encountered when using OGA for model selection and the estimation of segments containing change points in the cutting stage. The accuracy of multiple change point estimation is improved by applying sup-Wald-type test statistics in the refining stage. The proposed method successfully constructs confidence intervals for multiple change points by using a bootstrapping technique with a two-stage procedure to quantify the uncertainty of multiple change points. The reliable construction of confidence intervals makes this method a valuable addition to the field of change point analysis and regression modeling. The bootstrapping technique also guarantees asymptotic validity. Numerical studies demonstrate the statistical accuracy of the proposed method. Our method can also be applied together with block bootstrapping to the parameter changes of linear regression models with dependent errors.

## Figures and Tables

**Table 1:** Performance of different methods for multiple change point detection.

Method	$c_{\mathrm{all}}$		$c_{1}$	$c_{2}$	$c_{3}$
TSP_oga,wald_	90.70		98.50	96.80	97.80
		Mean	150.34	300.41	449.77
		SE	1.66	2.31	2.22
TSMCD_lasso_	72.60		95.20	95.80	96.40
		Mean	150.61	300.43	450.16
		SE	2.42	2.31	2.19

**Table 2:** Performance of bootstrap_oga,wald_.

(1−α)%	$a_{\mathrm{all}}$	$a_{1}$	$a_{2}$	$a_{3}$
90	93.80	91.80	93.80	91.00
95	96.80	95.80	95.80	95.60

**Table 3:** Bootstrap CIs for the change points.

Change Point	95% Bootstrap CIs	90% Bootstrap CIs
3074	[3072, 3084]	[3073, 3080]
3914	[3877, 3952]	[3882, 3948]

## Data Availability

The data presented in this study are available on request from the corresponding authors.

## Appendix A

**Proof** **of** **Theorem** **2.** We only sketch this proof because it is similar to the proof of Propositions 1 and 2 in [26]. To prove Theorem 2, we define $V\left(h\right)={\hat{\delta }}_{j;h}^{⊤}\left(Z_{j,h}^{⊤}M_{j}Z_{j,h}\right){\hat{\delta }}_{j;h}$. For the sake of simplicity, we denote $h_{0}=a_{j}$, which means that $h_{0}$ is the change point in $I_{k_{j}}$. If $h=h_{0}$, we can obtain that $Z_{j,h}=Z_{j,h_{0}}$, where $Z_{j,h_{0}}={\left(0,\cdots ,0,x_{h_{0}},\cdots ,x_{n_{j}^{\left(r\right)}}\right)}^{⊤}$. By Equation (9), ${\hat{a}}_{j,h}=\arg\max_{h}V\left(h\right)$. Note that

$$\begin{array}{cc} \; & {\hat{\delta }}_{j;h}={\left(Z_{j,h}^{⊤}M_{j}Z_{j,h}\right)}^{-1}\left(Z_{j,h}^{⊤}M_{j}Z_{j,h_{0}}\delta_{j}+Z_{j,h}^{⊤}M_{j}\varepsilon \right),\\ \; & {\hat{\delta }}_{j,h_{0}}=\delta_{j}+{\left(Z_{j,h_{0}}^{⊤}M_{j}Z_{j,h_{0}}\right)}^{-1}Z_{j,h_{0}}^{⊤}M_{j}\varepsilon . \end{array}$$

It follows that

$$\begin{array}{cc} V\left(h\right)-V\left(h_{0}\right) & =\delta_{j}^{⊤}\left\{\left(Z_{j,h_{0}}^{⊤}M_{j}Z_{j,h}\right){\left(Z_{j,h}^{⊤}M_{j}Z_{j,h}\right)}^{-1}\left(Z_{j,h}^{⊤}M_{j}Z_{j,h_{0}}\right)-\left(Z_{j,h_{0}}^{⊤}M_{j}Z_{j,h_{0}}\right)\right\}\delta_{j}\\ & \;+v(h), \end{array}$$

where

$$\begin{array}{cc} v(h) & =2\delta_{j}^{⊤}\left(Z_{j,h_{0}}^{⊤}M_{j}Z_{j,h}\right){\left(Z_{j,h}^{⊤}M_{j}Z_{j,h}\right)}^{-1}Z_{j,h_{0}}^{⊤}M_{j}\varepsilon -2\delta_{j}^{⊤}Z_{j,h_{0}}^{⊤}M_{j}\varepsilon \\ & \;+\varepsilon^{⊤}M_{j}Z_{j,h}{\left(Z_{j,h}^{⊤}M_{j}Z_{j,h}\right)}^{-1}Z_{j,h}^{⊤}M_{j}\varepsilon -\varepsilon^{⊤}M_{j}Z_{j,h_{0}}{\left(Z_{j,h_{0}}^{⊤}M_{j}Z_{j,h_{0}}\right)}^{-1}Z_{j,h_{0}}^{⊤}M_{j}\varepsilon . \end{array}$$

Define for $h\neq h_{0}$,

$$\begin{array}{cc} g(h) & =\delta_{j}^{⊤}\left|\left(Z_{j,h_{0}}^{⊤}M_{j}Z_{j,h}\right){\left(Z_{j,h}^{⊤}M_{j}Z_{j,h}\right)}^{-1}\left(Z_{j,h}^{⊤}M_{j}Z_{j,h_{0}}\right)\right.\\ & \;\left.-Z_{j,h_{0}}M_{j}Z_{j,h_{0}}\right|\delta_{j}/\left|h_{0}-h\right|. \end{array}$$

When $h=h_{0}$, define $g\left(h\right)=\delta_{j}^{⊤}\delta_{j}$. Then, we have

(A1) $$\begin{array}{cc} V\left(h\right)-V\left(h_{0}\right) & =-|h_{0}-h|g\left(h\right)+v\left(h\right). \end{array}$$

Let

$$Z_{\Delta }=\left\{\begin{array}{cc} Z_{j,h}-Z_{j,h_{0}}={\left(0,\cdots ,0,x_{h+1},\ldots ,x_{h_{0}},0,\ldots ,0\right)}^{⊤}, & h<h_{0},\\ Z_{j,h_{0}}-Z_{j,h}={\left(0,\cdots ,0,x_{h_{0}+1},\cdots ,x_{h},0,\ldots ,0\right)}^{⊤}, & h>h_{0},\\ 0, & h=h_{0}. \end{array}\right.$$

We have $Z_{j,h_{0}}=Z_{j,h}-Z_{\Delta }\mathrm{sgn}\left(h_{0}-h\right)$. It follows that

(A2) $$\begin{array}{cc} v(h) & =v_{1}\left(h\right)+v_{2}\left(h\right)+v_{3}\left(h\right)+v_{4}\left(h\right)+v_{5}\left(h\right)\\ & =\left[2\delta_{j}^{⊤}Z_{\Delta }^{⊤}\varepsilon \right]\mathrm{sgn}\left(h_{0}-h\right)\\ & \;-\left[2\delta_{j}^{⊤}Z_{\Delta }^{⊤}X_{j}{\left(X_{j}^{⊤}X_{j}\right)}^{-1}X_{j}^{⊤}\varepsilon \right]\mathrm{sgn}\left(h_{0}-h\right)\\ \; & \;-\left[2\delta_{j}^{⊤}\left(Z_{\Delta }^{⊤}M_{j}Z_{j,h}\right){\left(Z_{j,h}^{⊤}M_{j}Z_{j,h}\right)}^{-1}Z_{j,h}^{⊤}M_{j}\varepsilon \right]\mathrm{sgn}\left(h_{0}-h\right)\\ \; & \;+\varepsilon^{⊤}M_{j}Z_{j,h}{\left(Z_{j,h}^{⊤}M_{j}Z_{j,h}\right)}^{-1}Z_{j,h}^{⊤}M_{j}\varepsilon \\ & \;-\varepsilon^{⊤}M_{j}Z_{j,h_{0}}{\left(Z_{j,h_{0}}^{⊤}M_{j}Z_{j,h_{0}}\right)}^{-1}Z_{j,h_{0}}^{⊤}M_{j}\varepsilon . \end{array}$$

We now establish the convergence rate of the change point estimator ${\hat{a}}_{j,h}$ in Theorem 2. By Lemma A.2 in [26], there exists λ>0 such that for every ϵ>0, $\inf_{|h-h_{0}|>C∥\delta_{j}{∥}^{-2}}g\left(h\right)\geq \lambda {∥\delta_{j}∥}^{2}$ has a probability of at least 1−ϵ. Therefore, we only need to prove that

(A3) $$\begin{array}{cc} \; & P(|{\hat{a}}_{j,h}-a_{j}|>C∥\delta_{j}{∥}^{-2})\\ \leq & P\left(\underset{\left|h-h_{0}\right|>C{∥\delta_{j}∥}^{-2}}{\sup}V\left(h\right)\geq V\left(h_{0}\right)\right)\\ \leq & P\left(\underset{h\in K(C)}{\sup}\left|\frac{v(h)}{\left(h_{0}-h\right){∥\delta_{j}∥}^{2}}\right|>\lambda \right)\leq \epsilon , \end{array}$$

where $K\left(C\right)=\left\{h:\left|h-h_{0}\right|>C{∥\delta_{j}∥}^{-2}\;\mathrm{and}\;n_{j}^{\left(l\right)}+\eta N_{j}\leq h\leq n_{j}^{\left(r\right)}-\eta N_{j}\right\}$ for a small number of η>0. It is easy to show that

$$\begin{array}{cc} \underset{h\in K(C)}{\sup}\frac{|v_{2}\left(h\right)|}{|h_{0}-h|∥\delta_{j}{∥}^{2}} & \leq 2∥\delta_{j}{∥}^{-1}∥Z_{\Delta }^{⊤}x_{j}/\left(h_{0}-h\right)∥∥{\left(x_{j}^{⊤}x_{j}\right)}^{-1}x_{j}^{⊤}\varepsilon ∥\\ & =O_{p}(∥\delta_{j}{∥}^{-1}N_{j}^{-1/2})=o_{p}\left(1\right),\\ \underset{h\in K(C)}{\sup}\frac{v_{3}\left(h\right)}{|h_{0}-h|∥\delta_{j}{∥}^{2}} & \leq ∥\delta_{j}{∥}^{-1}∥\left(Z_{\Delta }^{⊤}M_{j}Z_{j,h}\right)/\left(h_{0}-h\right)∥∥{\left(Z_{j,h}^{⊤}M_{j}Z_{j,h}\right)}^{-1}Z_{j,h}^{⊤}M_{j}\varepsilon ∥\\ & =O_{p}(∥\delta_{j}{∥}^{-1}N_{j}^{-1/2})=o_{p}\left(1\right),\\ \underset{h\in K(C)}{\sup}\frac{|v_{4}\left(h\right)|}{|h_{0}-h|∥\delta_{j}{∥}^{2}} & \leq ∥\delta_{j}{∥}^{-2}∥{\left(Z_{j,h}^{⊤}M_{j}Z_{j,h}\right)}^{-1/2}Z_{j,h}^{⊤}M_{j}{\varepsilon ∥}^{2}/\left|h_{0}-h\right|\\ & =O_{p}(∥\delta_{j}{∥}^{-2}|h_{0}-h{|}^{-1})=O_{p}\left(1\right),\\ \underset{h\in K(C)}{\sup}\frac{|v_{5}\left(h\right)|}{|h_{0}-h|∥\delta_{j}{∥}^{2}} & \leq ∥\delta_{j}{∥}^{-2}∥{\left(Z_{j,h_{0}}^{⊤}M_{j}Z_{j,h_{0}}\right)}^{-1/2}Z_{j,h_{0}}^{⊤}M_{j}{\varepsilon ∥}^{2}/\left|h_{0}-h\right|\\ & =O_{p}(∥\delta_{j}{∥}^{-2}|h_{0}-h{|}^{-1})=O_{p}\left(1\right). \end{array}$$

By Lemma A.3 in [26], there exists a large *C* such that

$$\begin{array}{cc} & P\left(\underset{h\in K(C)}{\sup}\frac{|v_{1}\left(h\right)|}{|h_{0}-h|∥\delta_{j}{∥}^{2}}>\frac{\lambda }{5}\right)\\ \leq & P\left(\underset{h\in K(C)}{\sup}∥\delta_{j}{∥}^{-1}∥Z_{\Delta }^{⊤}\varepsilon /\left(h_{0}-h\right)∥>\frac{\lambda }{10}\right)\leq \frac{100B}{\lambda^{2}C}\leq \frac{\epsilon }{5}. \end{array}$$

This completes the proof of (A3). To study the limiting distribution of ${\hat{a}}_{j,h}$, we need to investigate the behavior of V(h) on $D\left(C\right)=\{h:|h-h_{0}|\leq C∥\delta_{j}{∥}^{-2}\}$. Since $∥X_{j}^{⊤}Z_{\Delta }∥=O_{p}(∥\delta_{j}{∥}^{-2})$ and $∥Z_{j,h}^{⊤}M_{j}Z_{\Delta }∥=O_{p}(∥\delta_{j}{∥}^{-2})$, we have

$$\begin{array}{cc} |h_{0}-h|g\left(h\right) & =\delta_{j}^{⊤}\left\{\left(Z_{j,h_{0}}^{⊤}M_{j}Z_{j,h}\right){\left(Z_{j,h}^{⊤}M_{j}Z_{j,h}\right)}^{-1}\left(Z_{j,h}^{⊤}M_{j}Z_{j,h_{0}}\right)-\left(Z_{j,h_{0}}^{⊤}M_{j}Z_{j,h_{0}}\right)\right\}\delta_{j}\\ \; & =\delta_{j}^{⊤}\left\{Z_{\Delta }^{⊤}Z_{\Delta }-Z_{\Delta }^{⊤}X_{j}{\left(X_{j}^{⊤}X_{j}\right)}^{-1}X_{j}^{⊤}Z_{\Delta }\right\}\delta_{j}\\ \; & \;-\delta_{j}\left\{\left(Z_{\Delta }^{⊤}M_{j}Z_{j,h}\right){\left(Z_{j,h}^{⊤}M_{j}Z_{j,h}\right)}^{-1}\left(Z_{j,h}^{⊤}M_{j}Z_{\Delta }\right)\right\}\delta_{j}\\ \; & =\delta_{j}^{⊤}Z_{\Delta }^{⊤}Z_{\Delta }\delta_{j}+o_{p}\left(1\right). \end{array}$$

Consider v(h) in (A2). It is straightforward to prove that if $|h-h_{0}|\leq C∥\delta_{j}{∥}^{-2}$, then $v_{2}\left(h\right)=O_{p}(∥\delta_{j}{∥}^{-1}N_{j}^{-1/2})=o_{p}\left(1\right)$, and $v_{3}\left(h\right)=O_{p}(∥\delta_{j}{∥}^{-1}N_{j}^{-1/2})=o_{p}\left(1\right)$. It can also be shown that

$$\begin{array}{cc} v_{4}\left(h\right)+v_{5}\left(h\right) & =\varepsilon^{⊤}M_{j}Z_{j,h_{0}}\left[{\left(Z_{j,h}^{⊤}M_{j}Z_{j,h}\right)}^{-1}-{\left(Z_{j,h_{0}}^{⊤}M_{j}Z_{j,h_{0}}\right)}^{-1}\right]Z_{j,h_{0}}^{⊤}M_{j}\varepsilon \\ & \;+\varepsilon^{⊤}M_{j}Z_{\Delta }{\left(Z_{j,h}^{⊤}M_{j}Z_{j,h}\right)}^{-1}Z_{j,h}^{⊤}M_{j}\varepsilon \\ & \;+\varepsilon^{⊤}M_{j}Z_{j,h_{0}}{\left(Z_{j,h}^{⊤}M_{j}Z_{j,h}\right)}^{-1}Z_{\Delta }^{⊤}M_{j}\varepsilon =o_{p}\left(1\right) \end{array}$$

on D(C). Therefore, we obtain that

(A4) $$V\left(h\right)-V\left(h_{0}\right)=-\delta_{j}^{⊤}Z_{\Delta }^{⊤}Z_{\Delta }\delta_{j}+\left[2\delta_{j}^{⊤}Z_{\Delta }^{⊤}\varepsilon \right]\mathrm{sgn}\left(h_{0}-h\right)+o_{p}\left(1\right).$$

In light of the proof of Proposition 2 in [26], we obtain the limiting distribution in Theorem 2. □

In the following, we introduce lemmas that are instrumental in proving Theorem 3.

**Lemma** **A1.** *If $\delta_{j}$ is fixed or $\delta_{j}\to 0$ but satisfies Assumption 5, then as $N_{j}\to \infty$,*

$${\mathrm{var}}^{*}\left(\varepsilon_{n_{j}^{\left(l\right)}}^{*}\right)=N_{j}^{-1}\sum_{i=n_{j}^{\left(l\right)}}^{n_{j}^{\left(r\right)}}{\left(\varepsilon_{i}-{\bar{\varepsilon }}_{N_{j}}\right)}^{2}+O_{p}\left(N_{j}^{-1}\right).$$

*where $\varepsilon_{n_{j}^{\left(l\right)}}^{*}$ is defined in* (11)*.*

**Proof** **f** **Lemma** **A1.** In view of (1) and (10), we have

(A5) $$\begin{array}{cc} {\tilde{\varepsilon }}_{i} & ={\left[x_{i}I\left(a_{j}<i\leq n_{j}^{\left(r\right)}\right)-\frac{1}{n_{j}^{\left(r\right)}-a_{j}}\sum_{l=a_{j}+1}^{n_{j}^{\left(r\right)}}x_{l}\right]}^{⊤}\delta_{j}+{\left(x_{i}-{\bar{x}}_{j}\right)}^{⊤}\left(\beta_{j}-{\hat{\beta }}_{j,h}\right)\\ \; & \;-{\left[x_{i}I\left({\hat{a}}_{j,h}<i\leq n_{j}^{\left(r\right)}\right)-\frac{1}{N_{j}}\sum_{l={\hat{a}}_{j,h}+1}^{n_{j}^{\left(r\right)}}x_{l}\right]}^{⊤}{\hat{\delta }}_{j,h}+\varepsilon_{i}-{\bar{\varepsilon }}_{N_{j}}, \end{array}$$

where ${\bar{x}}_{j}=\frac{1}{N_{j}}\sum_{l=n_{j}^{\left(l\right)}}^{n_{j}^{\left(r\right)}}x_{l}$ and ${\bar{\varepsilon }}_{N_{j}}=\frac{1}{N_{j}}\sum_{l=n_{j}^{\left(l\right)}}^{n_{j}^{\left(r\right)}}\varepsilon_{l}$. Assume $a_{j}<{\hat{a}}_{j,h}$ (the other case can be handled in a similar way). Since $E^{*}\left(\varepsilon_{n_{j}^{\left(l\right)}}^{*}\right)=N_{j}^{-1}\sum_{i=n_{j}^{\left(l\right)}}^{n_{j}^{\left(r\right)}}{\tilde{\varepsilon }}_{i}=0$, we have ${\mathrm{var}}^{*}\left(\varepsilon_{n_{j}^{\left(l\right)}}^{*}\right)=N_{j}^{-1}\sum_{i=n_{j}^{\left(l\right)}}^{n_{j}^{\left(r\right)}}{\tilde{\varepsilon }}_{i}^{2}$. It follows from (A5) that

(A6) $$\begin{array}{cc} {\mathrm{var}}^{*}\left(\varepsilon_{n_{j}^{\left(l\right)}}^{*}\right) & =\frac{1}{N_{j}}\sum_{i=n_{j}^{\left(l\right)}}^{n_{j}^{\left(r\right)}}{\left(\varepsilon_{i}-{\bar{\varepsilon }}_{N_{j}}\right)}^{2}+\frac{2}{N_{j}}\sum_{i=n_{j}^{\left(l\right)}}^{n_{j}^{\left(r\right)}}{\left(x_{i}-{\bar{x}}_{j}\right)}^{⊤}\left(\beta_{j}-{\hat{\beta }}_{j,h}\right)\left(\varepsilon_{i}-{\bar{\varepsilon }}_{N_{j}}\right)\\ \; & \;+\frac{1}{N_{j}}\sum_{i=n_{j}^{\left(l\right)}}^{n_{j}^{\left(r\right)}}{\left[{\left(x_{i}-{\bar{x}}_{j}\right)}^{⊤}\left(\beta_{j}-{\hat{\beta }}_{j,h}\right)\right]}^{2}+\frac{1}{N_{j}^{2}}{\left(\sum_{i={\hat{a}}_{j,h}+1}^{n_{j}^{\left(r\right)}}x_{i}^{⊤}\left(\delta_{j}-{\hat{\delta }}_{j,h}\right)\right)}^{2}\\ & \;+\frac{1}{N_{j}^{2}}{\left(\sum_{i=a_{j}+1}^{{\hat{a}}_{j,h}}x_{i}^{⊤}\delta_{j}\right)}^{2}+\frac{2}{N_{j}^{2}}\sum_{i=a_{j}+1}^{{\hat{a}}_{j,h}}x_{i}^{⊤}\delta_{j}\sum_{l={\hat{a}}_{j,h}+1}^{n_{j}^{\left(r\right)}}x_{l}^{⊤}\left(\delta_{j}-{\hat{\delta }}_{j,h}\right). \end{array}$$

According to Corollary 1 in [26], we have $\beta_{j}-{\hat{\beta }}_{j,h}=O_{p}\left(N_{j}^{-1/2}\right)$ and $\delta_{j}-{\hat{\delta }}_{j,h}=O_{p}\left(N_{j}^{-1/2}\right)$, which, combined with ${\hat{a}}_{j,h}=a_{j}+O_{p}(∥\delta_{j}{∥}^{-2})$, yields that $N_{j}^{-1}\sum_{i=a_{j}+1}^{{\hat{a}}_{j,h}}x_{i}^{⊤}\delta_{j}=O_{p}(N_{j}^{-1}∥\delta_{j}{∥}^{-1})$ and $N_{j}^{-1}\sum_{l={\hat{a}}_{j,h}+1}^{n_{j}^{\left(r\right)}}x_{l}^{⊤}\left(\delta_{j}-{\hat{\delta }}_{j,h}\right)=O_{p}\left(N_{j}^{-1/2}\right)$. This completes the proof. □

**Lemma** **A2.**
*For every ϵ>0,*

$$\underset{N_{j}\to \infty }{lim sup}P^{*}\left(\left|N_{j}^{-1}\sum_{i=n_{j}^{\left(l\right)}}^{n_{j}^{\left(r\right)}}{\left(\varepsilon_{i}^{*}\right)}^{2}-Var^{*}\left(\varepsilon_{n_{j}^{\left(l\right)}}^{*}\right)\right|\geq \epsilon \right)\leq \epsilon \sigma^{2}.$$

**Proof** **of** **Lemma** **A2.** We have for every ϵ>0 and every η>0,

(A7) $$\begin{array}{cc} \; & P^{*}\left(\left|N_{j}^{-1}\sum_{i=n_{j}^{\left(l\right)}}^{n_{j}^{\left(r\right)}}\varepsilon_{i}^{*2}-{\mathrm{var}}^{*}\left(\varepsilon_{n_{j}^{\left(l\right)}}^{*}\right)\right|\geq \epsilon \right)\\ = & P^{*}\left(\left|N_{j}^{-1}\sum_{i=n_{j}^{\left(l\right)}}^{n_{j}^{\left(r\right)}}\varepsilon_{i}^{*2}-N_{j}^{-1}\sum_{i=n_{j}^{\left(l\right)}}^{n_{j}^{\left(r\right)}}{\tilde{\varepsilon }}_{i}^{2}\right|\geq \epsilon \right)\\ \leq & P^{*}\left(\left|N_{j}^{-1}\sum_{i=n_{j}^{\left(l\right)}}^{n_{j}^{\left(r\right)}}\varepsilon_{i}^{*2}I(|\varepsilon_{i}^{*}|\geq \eta \sqrt{N_{j}})-N_{j}^{-1}\sum_{i=n_{j}^{\left(l\right)}}^{n_{j}^{\left(r\right)}}{\tilde{\varepsilon }}_{i}^{2}I(|{\tilde{\varepsilon }}_{i}\left|\geq \eta \sqrt{N_{j}}\right)\right|\geq \epsilon \right)\\ \; & +P^{*}\left(\left|N_{j}^{-1}\sum_{i=n_{j}^{\left(l\right)}}^{n_{j}^{\left(r\right)}}\varepsilon_{i}^{*2}I(|\varepsilon_{i}^{*}|<\eta \sqrt{N_{j}})-N_{j}^{-1}\sum_{i=n_{j}^{\left(l\right)}}^{n_{j}^{\left(r\right)}}{\tilde{\varepsilon }}_{i}^{2}I(|{\tilde{\varepsilon }}_{i}\left|<\eta \sqrt{N_{j}}\right)\right|\geq \epsilon \right) \end{array}$$

Clearly, for every ϵ>0 and every η>0, as $N_{j}\to \infty$, we have

$$N_{j}^{-1}\sum_{i=n_{j}^{\left(l\right)}}^{n_{j}^{\left(r\right)}}{\tilde{\varepsilon }}_{i}^{2}I(|{\tilde{\varepsilon }}_{i}\left|\geq \eta \sqrt{N_{j}}\right)\to 0\;a.s.$$

It follows that

$$P^{*}\left(N_{j}^{-1}\sum_{i=n_{j}^{\left(l\right)}}^{n_{j}^{\left(r\right)}}\varepsilon_{i}^{*2}I\left(\left|\varepsilon_{i}^{*}\right|\geq \eta \sqrt{N_{j}}\right)\geq \epsilon /2\right)\leq \frac{2}{\epsilon }N_{j}^{-1}\sum_{i=n_{j}^{\left(l\right)}}^{n_{j}^{\left(r\right)}}{\tilde{\varepsilon }}_{i}^{2}I\left(\left|{\tilde{\varepsilon }}_{i}\right|\geq \eta \sqrt{N_{j}}\right)\to 0,\;a.s.$$

Since

$$\begin{array}{cc} & \underset{N_{j}\to \infty }{lim sup}P^{*}\left(\left|N_{j}^{-1}\sum_{i=n_{j}^{\left(r\right)}}^{n_{j}^{\left(l\right)}}\varepsilon_{i}^{*2}I\left(\left|\varepsilon_{i}^{*}\right|<\eta \sqrt{N_{j}}\right)-N_{j}^{-1}\sum_{i=n_{j}^{\left(r\right)}}^{n_{j}^{\left(l\right)}}{\tilde{\varepsilon }}_{i}^{2}I\left(\left|{\tilde{\varepsilon }}_{i}\right|<\eta \sqrt{N_{j}}\right)\right|\geq \epsilon /2\right)\\ \leq & \frac{4}{\epsilon^{2}}\underset{N_{j}\to \infty }{lim sup}N_{j}^{-2}\sum_{i=n_{j}^{\left(r\right)}}^{n_{j}^{\left(l\right)}}{\tilde{\varepsilon }}_{i}^{4}I\left(\left|{\tilde{\varepsilon }}_{i}\right|<\eta \sqrt{N_{j}}\right)\\ \leq & \frac{4}{\epsilon^{2}}\eta^{2}\underset{n\to \infty }{lim sup}N_{j}^{-1}\sum_{i=n_{j}^{\left(r\right)}}^{n_{j}^{\left(l\right)}}{\tilde{\varepsilon }}_{i}^{2}=\frac{4}{\epsilon^{2}}\eta^{2}\sigma^{2}\;a.s.\;, \end{array}$$

choosing $\eta^{2}=\epsilon^{3}/8$ gives the assertion in Lemma A2. □

**Lemma** **A3.**
*Under the assumption that $\sum_{i=t_{1}}^{t_{2}}x_{i}x_{i}^{⊤}/\left(t_{1}-t_{2}\right)\to_{p}V$ as $t_{2}-t_{1}\to_{p}\infty$, for every ϵ>0, there exists λ>0 such that $g^{*}\geq \lambda {∥{\hat{\delta }}_{j,h}∥}^{2}$ with a probability of at least 1−ϵ, where $g^{*}=\inf_{\left|k_{2}-h\right|>N_{j}\eta }g^{*}\left(h_{b}\right)$.*

**Lemma** **A4.**
*Under the assumption that $\sum_{i=t_{1}}^{t_{2}}x_{i}x_{i}^{⊤}/\left(t_{1}-t_{2}\right)\to_{p}V$ as $t_{2}-t_{1}\to \infty$, for every ϵ>0 and λ>0, there exists $N_{0}>0$, such that $N_{j}>N_{0}$, $P^{*}\left(\right|h_{b}-h\left|>N_{j}\eta \right)<\epsilon$.*

The proofs of Lemmas A3 and A4 follow from [26] (see Lemmas A.2 and A.4). We next establish the consistency of ${\hat{a}}_{j,h_{b}}^{*}$ in Lemma A5, which serves as a key step in proving Theorem 3.

**Lemma** **A5.**
*If $\delta_{j}$ is fixed or $\delta_{j}\to 0$ but satisfies Assumption 5, under Assumptions 1–4, we have*

$${\hat{a}}_{j,h_{b}}^{*}-{\hat{a}}_{j,h}=O_{p}(∥{\hat{\delta }}_{j,h}{∥}^{-2}).$$

**Proof** **of** **Lemma** **A5.** We define $V^{*}\left(h_{b}\right)={\left({\hat{\delta }}_{j;h_{b}}^{*}\right)}^{⊤}\left(Z_{j,h_{b}}^{⊤}M_{j}Z_{j,h_{b}}\right){\hat{\delta }}_{j;h_{b}}^{*}$. By Equation (12), ${\hat{a}}_{j,h_{b}}^{*}=\arg\max_{h_{b}}V^{*}\left(h_{b}\right)$. Denote $h={\hat{a}}_{j,h}$. If $h_{b}=h$, then $Z_{j,h_{b}}=Z_{j,h}$. It can be seen from (11) that

$$\begin{array}{cc} & {\hat{\delta }}_{j;h_{b}}^{*}={\left(Z_{j,h_{b}}^{⊤}M_{j}Z_{j,h_{b}}\right)}^{-1}\left(Z_{j,h_{b}}^{⊤}M_{j}Z_{j,h}{\hat{\delta }}_{j,h}+Z_{j,h_{b}}^{⊤}M_{j}\varepsilon^{*}\right),\\ & {\hat{\delta }}_{j;h}^{*}={\hat{\delta }}_{j,h}+{\left(Z_{j,h}^{⊤}M_{j}Z_{j,h}\right)}^{-1}Z_{j,h}^{⊤}M_{j}\varepsilon^{*}. \end{array}$$

It follows that

$$\begin{array}{cc} V^{*}\left(h_{b}\right)-V^{*}\left(h\right)\; & ={\hat{\delta }}_{j,h}^{⊤}\left\{\left(Z_{j,h}^{⊤}M_{j}Z_{j,h_{b}}\right){\left(Z_{j,h_{b}}^{⊤}M_{j}Z_{j,h_{b}}\right)}^{-1}\left(Z_{j,h_{b}}^{⊤}M_{j}Z_{j,h}\right)-\left(Z_{j,h}^{⊤}M_{j}Z_{j,h}\right)\right\}{\hat{\delta }}_{j,h}\\ & \;+v^{*}\left(h_{b}\right), \end{array}$$

where

$$\begin{array}{cc} v^{*}\left(h_{b}\right) & =2{\hat{\delta }}_{j,h}^{⊤}\left(Z_{j,h}^{⊤}M_{j}Z_{j,h_{b}}\right){\left(Z_{j,h_{b}}^{⊤}M_{j}Z_{j,h_{b}}\right)}^{-1}Z_{j,h_{b}}^{⊤}M_{j}\varepsilon^{*}-2{\hat{\delta }}_{j,h}^{⊤}Z_{j,h}^{⊤}M_{j}\varepsilon^{*}\\ & \;+\varepsilon^{*⊤}M_{j}Z_{j,h_{b}}{\left(Z_{j,h_{b}}^{⊤}M_{j}Z_{j,h_{b}}\right)}^{-1}Z_{j,h_{b}}^{⊤}M_{j}\varepsilon^{*}-\varepsilon^{*⊤}M_{j}Z_{j,h}{\left(Z_{j,h}^{⊤}M_{j}Z_{j,h}\right)}^{-1}Z_{j,h}^{⊤}M_{j}\varepsilon^{*}. \end{array}$$

Define

$$g^{*}\left(h_{b}\right)=\left\{\begin{array}{cc} {\hat{\delta }}_{j,h}^{⊤}\left|\left(Z_{j,h}^{⊤}M_{j}Z_{j,h_{b}}\right){\left(Z_{j,h_{b}}^{⊤}M_{j}Z_{j,h_{b}}\right)}^{-1}\left(Z_{j,h_{b}}^{⊤}M_{j}Z_{j,h}\right)-Z_{j,h}M_{j}Z_{j,h}\right|{\hat{\delta }}_{j,h}/\left|h-h_{b}\right|, & \mathrm{if}\;h_{b}\neq h\\ {\hat{\delta }}_{j,h}^{⊤}{\hat{\delta }}_{j,h}, & \mathrm{if}\;h_{b}=h. \end{array}\right.$$

We have

(A8) $$V^{*}\left(h_{b}\right)-V^{*}\left(h\right)=-\left|h-h_{b}\right|g^{*}\left(h_{b}\right)+v^{*}\left(h_{b}\right).$$

Again, since $V^{*}\left(h_{b}\right)\geq V^{*}\left(h\right)$ by definition, it suffices to prove, by Lemma A4, that

$$P^{*}\left(\underset{\left|h_{b}-h\right|>C{∥{\hat{\delta }}_{j,h}∥}^{-2}}{\sup}V^{*}\left(h_{b}\right)\geq V^{*}\left(h\right)\right)<\epsilon .$$

By Lemmas A1 and A2, for any ϵ>0 and η>0, we have $P^{*}\left(\left|h_{b}-h\right|>N_{j}\eta \right)<\epsilon$ for a large $N_{j}$. Therefore, to prove the above equation, we only need to show that

$$P^{*}\left(\underset{h_{b}\in K^{*}\left(C\right)}{\sup}V\left(h_{b}\right)\geq V\left(h\right)\right)<\epsilon ,$$

where $K^{*}\left(C\right)=\left\{h_{b}:\left|h_{b}-h\right|>C{∥{\hat{\delta }}_{j,h}∥}^{-2}\;and\;n_{j}^{\left(l\right)}+N_{j}\eta \leq h_{b}\leq n_{j}^{\left(r\right)}-\eta N_{j}\right\}$ for a small number η>0. Finding $\sup_{h_{b}\in K^{*}\left(C\right)}V\left(h_{b}\right)$ is equivalent to a restricted search, this is legitimate only after the consistency has been established. Thus, by Lemma A3, it suffices to show that

(A9) $$P^{*}\left(\underset{h_{b}\in K^{*}\left(C\right)}{\sup}\frac{|v^{*}\left(h_{b}\right)|}{|h-h_{b}|∥{\hat{\delta }}_{j,h}{∥}^{2}}>\lambda \right)<\epsilon .$$

Next, consider $v^{*}\left(h_{b}\right)$. Denote

$$Z_{\Delta_{b}}=\left\{\begin{array}{cc} Z_{j,h_{b}}-Z_{j,h}={\left(0,\cdots ,0,x_{h_{b}+1},\ldots ,x_{h},0,\ldots ,0\right)}^{⊤}, & h_{b}<h,\\ Z_{j,h}-Z_{j,h_{b}}={\left(0,\cdots ,0,x_{h+1},\cdots ,x_{h_{b}},0,\ldots ,0\right)}^{⊤}, & h_{b}>h,\\ 0, & h_{b}=h. \end{array}\right.$$

It follows that $Z_{j,h_{b}}=Z_{j,h}+Z_{\Delta_{b}}\mathrm{sgn}\left(h-h_{b}\right)$. Thus, we have

(A10) $$\begin{array}{cc} v^{*}\left(h_{b}\right) & =v_{1}^{*}\left(h_{b}\right)+v_{2}^{*}\left(h_{b}\right)+v_{3}^{*}\left(h_{b}\right)+v_{4}^{*}\left(h_{b}\right)+v_{5}^{*}\left(h_{b}\right)\\ & =\left[2{\hat{\delta }}_{j,h}^{⊤}Z_{\Delta_{b}}^{⊤}\varepsilon^{*}\right]\mathrm{sgn}\left(h-h_{b}\right)\\ & \;-\left[2{\hat{\delta }}_{j,h}^{⊤}Z_{\Delta_{b}}^{⊤}x_{j}{\left(x_{j}^{⊤}x_{j}\right)}^{-1}x_{j}^{⊤}\varepsilon^{*}\right]\mathrm{sgn}\left(h-h_{b}\right)\\ \; & \;-\left[2{\hat{\delta }}_{j,h}^{⊤}\left(Z_{\Delta_{b}}^{⊤}M_{j}Z_{j,h_{b}}\right){\left(Z_{j,h_{b}}^{⊤}M_{j}Z_{j,h_{b}}\right)}^{-1}Z_{j,h_{b}}^{⊤}M_{j}\varepsilon^{*}\right]\mathrm{sgn}\left(h-h_{b}\right)\\ \; & \;+\varepsilon^{*⊤}M_{j}Z_{j,h_{b}}{\left(Z_{j,h_{b}}^{⊤}M_{j}Z_{j,h_{b}}\right)}^{-1}Z_{j,h_{b}}^{⊤}M_{j}\varepsilon^{*}\\ & \;-\varepsilon^{*⊤}M_{j}Z_{j,h}{\left(Z_{j,h}^{⊤}M_{j}Z_{j,h}\right)}^{-1}Z_{j,h}^{⊤}M_{j}\varepsilon^{*}. \end{array}$$

By Lemmas A1 and A2, we deduce that ${\left(X_{j}^{⊤}X_{j}\right)}^{-1}X_{j}^{⊤}\varepsilon^{*}=N_{j}^{-1/2}O_{p^{*}}\left(1\right).$ Since $N_{j}^{-1/2+\alpha }\delta_{j}^{-1}\to 0$ and

$$N_{j}^{-1/2+\alpha }\delta_{j}^{-1}-N_{j}^{-1/2+\alpha }{\hat{\delta }}_{j,h}^{-1}=N_{j}^{-1/2+\alpha }\delta_{j}^{-1}\left({\hat{\delta }}_{j,h}-\delta_{j}\right){\hat{\delta }}_{j,h}^{-1}=O_{p^{*}}\left(N_{j}^{-1+\alpha }\delta_{j}^{-2}\right),$$

by Corollary 1 in [26], we have $N_{j}^{-1/2+\alpha }{\hat{\delta }}_{j,h}^{-1}$ tends to zero. It follows from $Z_{\Delta_{b}}^{⊤}X_{j}=\left|h-h_{b}\right|O_{p}\left(1\right)$ that

$$\begin{array}{cc} \underset{h_{b}\in K^{*}\left(C\right)}{\sup}\frac{|v_{2}^{*}\left(h_{b}\right)|}{|h-h_{b}|∥{\hat{\delta }}_{j,h}{∥}^{2}} & \leq 2∥{\hat{\delta }}_{j,h}{∥}^{-1}∥Z_{\Delta_{b}}^{⊤}X_{j}/\left(h-h_{b}\right)∥∥{\left(X_{j}^{⊤}X_{j}\right)}^{-1}X_{j}^{⊤}\varepsilon^{*}∥\\ & =O_{p^{*}}(∥{\hat{\delta }}_{j,h}{∥}^{-1}N_{j}^{-1/2})=o_{p^{*}}\left(1\right). \end{array}$$

Similarly, we have $∥\left(Z_{\Delta_{b}}^{⊤}M_{j}Z_{j,h_{b}}\right)∥=|h-h_{b}|O_{p^{*}}\left(1\right)$, and ${\left(Z_{j,h_{b}}^{⊤}M_{j}Z_{j,h_{b}}\right)}^{-1}Z_{j,h_{b}}^{⊤}M_{j}\varepsilon^{*}=N_{j}^{-1/2}O_{p^{*}}\left(1\right)$ uniformly on $K^{*}\left(C\right)$, which implies

$$\begin{array}{cc} \underset{h_{b}\in K^{*}\left(C\right)}{\sup}\frac{v_{3}\left(h_{b}\right)}{|h-h_{b}|∥{\hat{\delta }}_{j,h}{∥}^{2}} & \leq ∥{\hat{\delta }}_{j,h}{∥}^{-1}∥\left(Z_{\Delta_{b}}^{⊤}M_{j}Z_{j,h_{b}}\right)/\left(h-h_{b}\right)∥∥{\left(Z_{j,h_{b}}^{⊤}M_{j}Z_{j,h_{b}}\right)}^{-1}Z_{j,h_{b}}^{⊤}M_{j}\varepsilon^{*}∥\\ & =O_{p^{*}}(∥{\hat{\delta }}_{j,h}{∥}^{-1}N_{j}^{-1/2})=o_{p^{*}}\left(1\right). \end{array}$$

Furthermore, it is easy to show that the expressions $\sup_{h_{b}\in K^{*}\left(C\right)}\frac{|v_{4}^{*}\left(h_{b}\right)|}{|h-h_{b}|∥{\hat{\delta }}_{j,h}{∥}^{2}}$ and $\sup_{h_{b}\in K^{*}\left(C\right)}\frac{|v_{5}^{*}\left(h\right)|}{|h-h_{b}|∥{\hat{\delta }}_{j,h}{∥}^{2}}$ are uniformly $O_{p^{*}}\left(1\right)$ on $K^{*}\left(C\right)$. By Lemma A.3 in [26],

$$\begin{array}{cc} & P^{*}\left(\underset{h_{b}\in K^{*}\left(C\right)}{\sup}\frac{|v_{1}^{*}\left(h_{b}\right)|}{|h-h_{b}|∥{\hat{\delta }}_{j,h}{∥}^{2}}>\frac{\lambda }{5}\right)\\ \leq & P^{*}\left(\underset{h_{b}\in K^{*}\left(C\right)}{\sup}∥{\hat{\delta }}_{j,h}{∥}^{-1}∥Z_{\Delta_{b}}^{⊤}\varepsilon^{*}/\left(h-h_{b}\right)∥>\frac{\lambda }{10}\right)\leq \frac{100B}{\lambda^{2}C}\leq \frac{\epsilon }{5} \end{array}$$

for large *C*. In conclution, the consistency of the bootstrap estimator has been established. □

**Proof** **of** **Theorem** **3.** Lemma A5 implies that when *C* is large, ${\hat{a}}_{j,h_{b}}^{*}$ has a high probability of being outside the set $K^{*}\left(C\right)$. Let $D^{*}\left(C\right)$ denote the complement set of $K^{*}\left(C\right)$ such that $D^{*}\left(C\right)=\{h_{b}:|h_{b}-h|\leq C∥{\hat{\delta }}_{j,h}{∥}^{-2}\}$. To study the limiting distribution, we consider the expression of $V^{*}\left(h_{b}\right)-V^{*}\left(h\right)$ on $D^{*}\left(C\right)$. We use $Z_{j,h}=Z_{j,h_{b}}-Z_{\Delta_{b}}\mathrm{sgn}\left(h-h_{b}\right)$ to obtain that

(A11) $$\begin{array}{cc} \left|h-h_{b}\right|g^{*}\left(h_{b}\right) & ={\hat{\delta }}_{j,h}^{⊤}\left\{Z_{j,h}M_{j}Z_{j,h}-Z_{j,h}^{⊤}M_{j}Z_{j,h_{b}}{\left(Z_{j,h_{b}}M_{j}Z_{j,h_{b}}\right)}^{-1}Z_{j,h_{b}}^{⊤}M_{j}Z_{j,h}\right\}{\hat{\delta }}_{j,h}\\ \; & ={\hat{\delta }}_{j,h}^{⊤}\left\{Z_{\Delta_{b}}^{⊤}Z_{\Delta_{b}}-Z_{\Delta_{b}}^{⊤}X_{j}{\left(X_{j}^{⊤}X_{j}\right)}^{-1}X_{j}^{⊤}Z_{\Delta_{b}}\right\}{\hat{\delta }}_{j,h}\\ & \;-{\hat{\delta }}_{j,h}^{⊤}Z_{\Delta_{b}}^{⊤}M_{j}Z_{j,h_{b}}{\left(Z_{j,h_{b}}M_{j}Z_{j,h_{b}}\right)}^{-1}Z_{j,h_{b}}^{⊤}M_{j}Z_{\Delta_{b}}{\hat{\delta }}_{j,h}. \end{array}$$

Since $∥X_{j}^{⊤}Z_{\Delta_{b}}∥=O_{p^{*}}(∥{\hat{\delta }}_{j,h}{∥}^{-2})$ and $∥Z_{j,h_{b}}^{⊤}M_{j}Z_{\Delta_{b}}∥=O_{p^{*}}(∥{\hat{\delta }}_{j,h}{∥}^{-2})$, we have

$$|h-h_{b}|g^{*}\left(h_{b}\right)={\hat{\delta }}_{j,h}^{⊤}Z_{\Delta_{b}}^{⊤}Z_{\Delta_{b}}{\hat{\delta }}_{j,h}+o_{p^{*}}\left(1\right).$$

Since $\left|h-h_{b}\right|\leq C{∥{\hat{\delta }}_{j,h}∥}^{-2}$, we deduce that $v_{2}^{*}\left(h_{b}\right)$ and $v_{3}^{*}\left(h_{b}\right)$ are both bounded by $O_{p^{*}}(N_{j}^{-1/2}∥{\hat{\delta }}_{j,h}{∥}^{-1})=o_{p^{*}}\left(1\right)$, and $v_{4}^{*}\left(h_{b}\right)$ and $v_{5}^{*}\left(h_{b}\right)$ are both bounded by $o_{p^{*}}\left(1\right)$ uniformly on $D^{*}\left(C\right)$. By the above results, we obtain that

$$\begin{array}{cc} V^{*}\left(h_{b}\right)-V^{*}\left(h\right) & =-{\hat{\delta }}_{j,h}^{⊤}Z_{\Delta_{b}}^{⊤}Z_{\Delta_{b}}{\hat{\delta }}_{j,h}+2{\hat{\delta }}_{j,h}^{⊤}Z_{\Delta_{b}}^{⊤}\varepsilon^{*}\mathrm{sgn}\left(h-h_{b}\right)+o_{p^{*}}\left(1\right), \end{array}$$

which combined with (A4) completes the proof. □
